# Powassan and Eastern Equine Encephalitis Virus Seroprevalence in Endemic Areas, United States, 2019–2020

**DOI:** 10.3201/eid3105.240893

**Published:** 2025-05

**Authors:** Hannah Padda, Claire Y.-H. Huang, Kacie Grimm, Brad J. Biggerstaff, Jeremy P. Ledermann, Janae Raetz, Karen Boroughs, Eric C. Mossel, Stacey W. Martin, Jennifer A. Lehman, Rebecca L. Townsend, David Krysztof, Paula Saá, Emily T.N. Dinh, Mary Grace Stobierski, Brenda Esponda-Morrison, Karen Ann A. Wolujewicz, Matthew Osborne, Catherine M. Brown, Brandi Hopkins, Elizabeth K. Schiffman, Alex Garvin, Xia Lee, Rebecca A. Osborn, Ryan J. Wozniak, Aaron C. Brault, Sridhar V. Basavaraju, Susan L. Stramer, J. Erin Staples, Carolyn V. Gould

**Affiliations:** Author affiliations: Centers for Disease Control and Prevention, Fort Collins, Colorado, USA (H. Padda, C.Y.-H. Huang, B.J. Biggerstaff, J.P. Ledermann, J. Raetz, K. Boroughs, E.C. Mossel, S.W. Martin, J.A. Lehman, A.C. Brault, J.E. Staples, C.V. Gould); American Red Cross, Rockville, Maryland, USA (K. Grimm, R.L. Townsend, D. Krysztof, P. Saá); Michigan Department of Health and Human Services, Lansing, Michigan, USA (E.T.N. Dinh, M.G. Stobierski); Connecticut Department of Public Health, Hartford, Connecticut, USA (B. Esponda-Morrison, K.A.A. Wolujewicz); Massachusetts Department of Public Health, Boston, Massachusetts, USA (M. Osborne, C.M. Brown, B. Hopkins); Minnesota Department of Health, Saint Paul, Minnesota, USA (E.K. Schiffman, A. Garvin); Wisconsin Department of Health Services, Madison, Wisconsin, USA (X. Lee, R.A. Osborn, R.J. Wozniak); Centers for Disease Control and Prevention, Atlanta, Georgia, USA (S.V. Basavaraju); Infectious Disease Consultant, North Potomac, Maryland, USA (S.L. Stramer)

**Keywords:** Powassan virus, Eastern equine encephalitis virus, vector-borne infections, viruses, blood donor, serosurvey, seroprevalence, blood safety, organ transplantation, United States

## Abstract

Powassan virus (POWV) and Eastern equine encephalitis virus (EEEV) are regionally endemic arboviruses in the United States that can cause neuroinvasive disease and death. Recent identification of EEEV transmission through organ transplantation and POWV transmission through blood transfusion have increased concerns about infection risk. After historically high numbers of cases of both viruses were reported in 2019, we conducted a seroprevalence survey using blood donation samples from selected endemic counties. Specimens were screened for virus-specific neutralizing antibodies, and population seroprevalence was estimated using weights calibrated to county population census data. For POWV, median county seroprevalence in 4 states was 0.84%, ranging from 0% (95% CI 0%–2.28%) to 11.5% (95% CI 0.82%–40.9%). EEEV infection was identified in a single county (estimated seroprevalence 1.62% [95% CI 0.04%–8.75%]). Although seroprevalence estimates in sampled areas were generally low, additional investigation of higher-prevalence areas could inform risk for transmission from asymptomatic blood and organ donors.

Powassan virus (POWV) and Eastern equine encephalitis virus (EEEV) are geographically focal arthropodborne viruses (arboviruses) in the United States (*1*–*3*). Most human infections are asymptomatic, but both viruses can cause disease ranging from acute febrile illness to severe encephalitis that can cause long-term disability or death. Recent increases in disease cases, outbreaks with high rates of illness and deaths, and identification of blood and organ donor–transmitted infections have led to greater concerns about human risk.

POWV, a flavivirus in the tickborne encephalitis serogroup, is spread to humans primarily by *Ixodes* spp. ticks in eastern Canada and the upper Midwest and Northeast United States (*2*). The number of POWV disease cases reported to the Centers for Disease Control and Prevention (CDC) has been rising; an average of 10 cases were reported annually before 2016, compared with 30 cases reported annually during 2016–2022 (*4*). In 2018, a probable case of blood transfusion transmission of POWV from a Wisconsin donor was identified in a kidney transplant recipient with neuroinvasive disease (*5*).

EEEV is an alphavirus spread to humans by several species of mosquitoes, most often near freshwater hardwood swamps in US states of the Atlantic, Gulf Coast, and Great Lakes regions (*6*). EEEV disease has the highest reported case-fatality rate among arboviral diseases endemic to the United States; 78 (41%) deaths were reported among 189 neurologic cases of EEEV disease during 2003–2022 (*7*). In 2017, EEEV disease developed in 3 organ transplant recipients who received an organ from an infected donor, and 2 died (*8*). In 2019, a record number of EEEV disease cases was reported during a multistate outbreak of 34 cases in 7 states with 12 (35%) fatalities (*9*,*10*).

Few POWV and EEEV seroprevalence studies have been performed to assess the burden of infection. We conducted a seroprevalence study using residual blood donation samples collected from persons residing in selected POWV- and EEEV-endemic areas during 2019–2020 to determine infection risk among county residents and to assess potential risk to the blood supply for these pathogens.

## Methods

### Ethics Considerations

Routine informed consent obtained at the time of blood donation includes potential use of samples and demographic information for research purposes. The protocol for this study was approved by the American Red Cross Institutional Review Board (protocol no. 2021-038).

### Study Population

We obtained residual serum and plasma samples from blood donations collected by the American Red Cross during December 2019–July 2020 from a selected number of states and counties. We restricted the study population to unique blood donors (all >16 years of age) who resided in a county endemic for either POWV or EEEV, which we defined as having either >2 human disease cases in 2019 or 1 case in 2019 and >1 case during 2010–2018 reported to CDC’s ArboNET, the national arboviral disease surveillance system. We designed the criteria to capture counties with suitable habitats for the sustained circulation of the viruses resulting in human disease cases; however, we limited counties assessed to those with available blood donor samples.

### Sampling Strategy

We selected samples using proportional-to-size stratified sampling by county. We specified the expected seroprevalences and acceptable margins of error (ME) on the basis of the only known previously published seroprevalence estimates, both from focal areas of New Jersey that experienced outbreaks of human POWV disease in 2019 (*11*) and EEEV disease in 1959 (*12*). For POWV, the expected seroprevalence was 0.5% and the ME 0.4%. Given the low expected seroprevalence, we used the available blood donor population, rather than the county population, to calculate sample sizes. For EEEV, the expected seroprevalence was 3% and the ME 2%. We used the 2020 United States Census Bureau County population of adults to determine the sample size needed to calculate EEEV population seroprevalence (*13*). We randomly chose samples from the available pool of donor samples in each county.

### Laboratory Testing

We first screened samples for the presence of neutralizing antibodies against POWV, EEEV, or both, depending on the endemic county (Table 1). For initial POWV screening, we used a reporter virus–based microfocus neutralization reduction test (R-mFRNT) to identify positive samples as those with a 90% R-mFRNT (R-mFRNT_90_) titer >10. For EEEV, we used plaque reduction neutralization test (PRNT) to identify positive samples as those with a 90% PRNT (PRNT_90_) titer >10 (*14*). The high-throughput R-mFRNT method is based on the same principle as PRNT in measuring virus infection foci (plaques) reduction by neutralizing antibodies (*15*). The method uses live reporter–POWV and reporter–West Nile virus (WNV) that were engineered using the chimeric platform previously described (*16*). We validated the R-mFRNT_90_ using reporter viruses against PRNT_90_ using wild-type viruses with panels of positive POWV or WNV samples before use in this study and found strong correlation of the 90% effective concentrations between the R-mFRNT_90_ and PRNT_90_ assays.

**Table 1:** Demographics of blood donors tested for POWV and EEEV and disease cases and population census of selected counties of residence in study of seroprevalence in endemic areas, United States, 2019–2020*

Demographics	No. disease cases reported, 2010–2019		2020 US Census population, age >15 y	No. (%) specimens tested	
	POWV	EEEV		POWV	EEEV
Sex					
F				835 (47.2)	276 (48.7)
M				935 (52.8)	291 (51.3)
Age group, y					
16–29				225 (12.7)	65 (11.5)
30–49				436 (24.6)	147 (25.9)
50–64				751 (42.4)	243 (42.9)
>65				358 (20.2)	112 (19.8)
State/county of residence					
Connecticut					
Fairfield	3		771,950	420	
Litchfield	2		155,110	164	
New London		3	224,538		17
Massachusetts					
Barnstable	5		187,694	21	
Bristol		4	467,915		36
Essex	9	3	650,007	184	49
Middlesex	10	3	1,343,808	337	100
Norfolk	2		583,823	206	
Plymouth		4	428,646		32
Worcester	2	4	684,405	160	52
Michigan					
Berrien		2	126,184		81
Cass		2	42,929		24
Kalamazoo		5	216,792		137
Van Buren		2	60,928		39
Minnesota					
Anoka	5		284,136	141	
Cass	2		24,286	3	
Itasca	7		37,544	8	
Morrison	3		26,551	8	
Wisconsin					
Jackson	2		16,881	16	
Shawano	2		33,692	3	
Trempealeau	2		23,357	19	
Wood	2		59,811	80	
Total				1,770	567

*Blank cells indicate not applicable. EEEV, Eastern equine encephalitis virus; POWV, Powassan virus.

We also endpoint titrated samples that screened positive for POWV neutralizing antibodies for both POWV and WNV by R-mFRNT_90_ to assess potential cross-reactivity between the 2 flaviviruses. We conducted the endpoints of R-mFRNT_90_ in 2-fold serial dilutions of samples in triplicate to determine the effective concentration for 90% neutralization (EC_90_; concentration is the log_10_ reciprocal of dilutions) by the 4-parameter logistic curve analysis using GraphPad Prism version 10.1.2 (GraphPad Software Inc., https://www.graphpad.com). We used a >4-fold difference in the R-mFRNT_90_ to confirm exposure to POWV or WNV. We considered similar titers (<4-fold difference) to both viruses as undifferentiated flavivirus exposures and did not include them in POWV seroprevalence estimates. We then tested samples positive for POWV- and EEEV-neutralizing antibodies for presence of IgM using IgM capture ELISA (MAC-ELISA) for POWV and a microsphere immunoassay for EEEV to assess for evidence of recent infection, as previously described (*17*,*18*).

### Statistical Analysis

We calculated seroprevalence estimates and 95% CIs at the county population level by calibrating the sample design weights to population age group distributions obtained from the 2020 US Census Bureau data (*13*). We calibrated sample weights using poststratification to the census data on the basis of the age group of the blood donors and county population. For the weighting calibration, we grouped age into 4 categories (Table 1) according to previously described methods (*19*). We computed estimates both for presence of neutralizing antibodies (any previous infection) and for presence of both neutralizing antibodies and IgM (recent infection) (*20*). We excluded counties with <5 blood donor samples because of instability in the estimates. For the county in which the source of infection was most likely outside the county of residence according to previous case investigations by the state health department, we restricted seroprevalence estimates to the county blood donor population, rather than to the general population.

To estimate the percentage of EEEV infections that resulted in neuroinvasive disease, we multiplied estimated county IgM seroprevalence (with 95% CI) by the county population (>15 years of age, the closest available census data age category to the blood donor population) for the expected number of recent infections. We then divided the reported number of EEEV disease cases during June 2019–July 2020 by the expected number of recent infections (95% CI). We assumed IgM against EEEV persisted for up to 6 months for this calculation (*21*,*22*). We analyzed data using R version 4.3.1 using the survey package version 4.2 (*23*).

## Results

### POWV Seroprevalence

**Figure 1:**
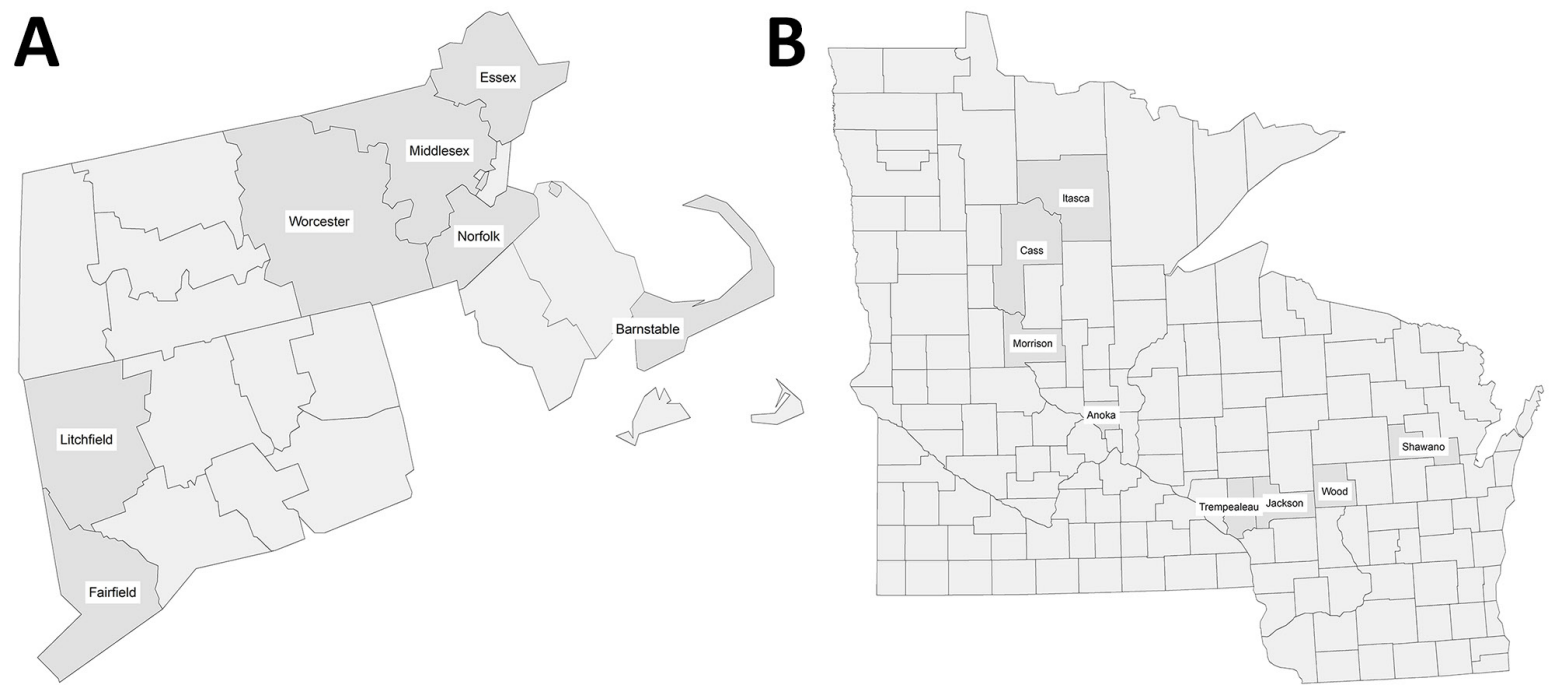
Selected counties for Powassan virus sampling in in study of Powassan virus and Eastern equine encephalitis virus seroprevalence in endemic areas, United States, 2019–2020. A) Connecticut and Massachusetts; B) Minnesota and Wisconsin.

We tested a total of 1,770 samples from 15 counties in 4 states (Connecticut, Massachusetts, Minnesota, and Wisconsin) for evidence of POWV infection (Table 1; Figure 1). We found 50 (2.8%) samples had neutralizing antibodies for either POWV or WNV; 3 of those samples had WNV-specific neutralizing antibodies and 31 had indistinguishable results. Sixteen samples had POWV-specific neutralizing antibodies: 4 from Connecticut, 5 from Massachusetts, 3 from Minnesota, and 4 from Wisconsin (Table 2). County estimates by state among counties with locally acquired POWV infections ranged from 0% (95% CI 0%–2.28%) to 11.5% (95% CI 0.82%–40.9%). The highest and almost equivalent estimates were in 2 neighboring counties in Wisconsin: 11.5% (95% CI 0.82%–40.9%) and 11.5% (95% CI 0.87%–40.3%). Of the 16 samples with POWV-specific neutralizing antibodies, 6 (38%) were IgM positive. Recent seroprevalence estimates by county ranged from 0% (95% CI 0%–2.28%) to 1.68% (95% CI 0.14%–6.70%) (Table 2). In Anoka County, Minnesota, where cases were considered likely to be travel-associated, estimated county blood donor seroprevalence was 1.42% (95% CI 0.39%–5.02%) for any infection and 0.71% (95% CI 0.04%–3.91%) for recent infection (Table 2).

**Table 2:** Blood donor and estimated population seroprevalence for Powassan virus in selected endemic counties in study of Powassan virus and Eastern equine encephalitis virus seroprevalence in endemic areas, United States, 2019–2020

	Calculated sample size	No. samples tested	No. (%) donors		% Seroprevalence (95% CI)	
			Neutralizing antibody positive	Neutralizing antibody and IgM positive		
					Estimated*	Estimated recent†
Connecticut						
Fairfield	422	420	2 (0.48)	1 (0.24)	0.29 (0.04–1.04)	0.15 (0.00–0.81)
Litchfield	164	164	2 (1.2)	2 (1.2)	1.68 (0.14–6.70)	1.68 (0.14–6.63)
Massachusetts						
Barnstable	21	21	0	0	0 (0–16.1)	0 (0–16.1)
Essex	184	184	3 (1.6)	2 (1.1)	1.12 (0.22–3.32)	0.81 (0.09–2.96)
Middlesex	339	337	2 (0.59)	0	0.98 (0.10–3.67)	0 (0–1.09)
Norfolk	206	206	1 (0.49)	0	0.70 (0.02–3.81)	0 (0–1.77)
Worcester	160	160	0	0	0 (0–2.28)	0 (0–2.28)
Minnesota						
Anoka‡	141	141	2 (1.4)	1 (0.71)	1.42 (0.39–5.02)	0.71 (0.04–3.91)
Cass§	3	3	0	0	–	–
Itasca	8	8	0	0	0 (0–36.9)	0 (0–36.9)
Morrison	8	8	0	0	0 (0–36.9)	0 (0–36.9)
Wisconsin						
Jackson	16	16	1 (6.3)	0	11.48 (0.82–40.92)	0 (0–20.59)
Shawano§	3	3	0	0	–	–
Trempealeau	19	19	1 (5.3)	0	11.47 (0.87–40.3)	0 (0–17.65)
Wood	78	80	2 (2.5)	0	2.12 (0.27–7.37)	0 (0–4.51)

*Neutralizing antibodies present. †Neutralizing antibodies and IgM present. ‡County where cases were suspected to be travel-associated rather than locally acquired. §Counties with <5 samples were excluded from seroprevalence calculations.

### EEEV Seroprevalence

**Figure 2:**
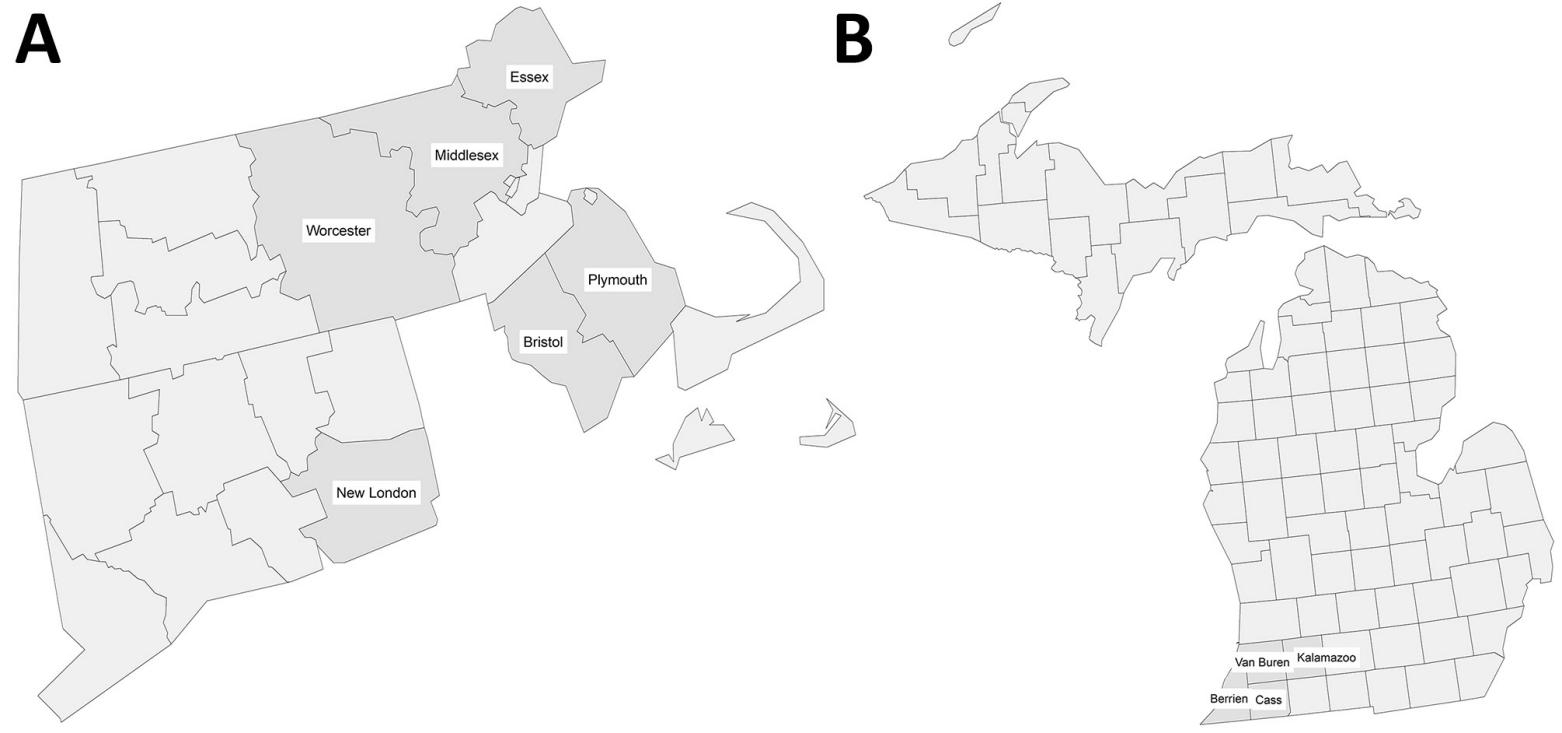
Selected counties for Eastern equine encephalitis virus sampling in study of Powassan virus and Eastern equine encephalitis virus seroprevalence in endemic areas, United States, 2019–2020. A) Connecticut and Massachusetts; B) Michigan.

We tested a total of 567 samples from 10 counties in 3 states (Connecticut, Massachusetts, and Michigan) for evidence of EEEV infection (Table 1; Figures 2). Only 1 sample in Worcester County, Massachusetts, was positive for both neutralizing antibodies and IgM against EEEV, for an estimated recent infection seroprevalence of 1.62% (95% CI 0.04%–8.75%) (Table 3). The demographics of this blood donor did not match any of the EEEV disease case-patients reported to ArboNET; therefore, the donor most likely had an asymptomatic infection or nonneuroinvasive disease that was not diagnosed. The estimated total number of recent infections in Worcester was 11,086 (95% CI 272–59,912), and 3 human EEEV neuroinvasive disease cases were reported in the county in 2019, for a neuroinvasive disease–to-infection percentage of 0.027% (95% CI 0.005%–1.10%).

**Table 3:** Blood donor and estimated population seroprevalence for Eastern equine encephalitis virus in selected counties in study of Powassan virus and Eastern equine encephalitis virus seroprevalence in endemic areas, United States, 2019–2020

State/county	Calculated sample size	No. samples tested	No. (%) donors				
			Neutralizing antibody positive	Neutralizing antibody and IgM positive		% Seroprevalence (95% CI)	
						Estimated*	Estimated recent†
Connecticut							
New London	17	17	0	0		0 (0–19.5)	0 (0–19.5)
Massachusetts							
Bristol	36	36	0	0		0 (0–9.74)	0 (0–9.74)
Essex	49	49	0	0		0 (0–7.25)	0 (0–7.25)
Middlesex	100	100	0	0		0 (0–3.62)	0 (0–3.62)
Plymouth	32	32	0	0		0 (0–10.9)	0 (0–10.9)
Worcester	52	52	1 (1.9)	1 (1.9)		1.62 (0.04–8.75)	1.62 (0.04–8.75)
Michigan							
Berrien	81	81	0	0		0 (0–4.45)	0 (0–4.45)
Cass	27	24	0	0		0 (0–14.3)	0 (0–14.3)
Kalamazoo	137	137	0	0		0 (0–2.66)	0 (0–2.66)
Van Buren	39	39	0	0		0 (0–9.03)	0 (0–9.03)

*Neutralizing antibodies present. †Neutralizing antibodies and IgM present.

## Discussion

On the basis of this blood donor serosurvey, we estimated that population seroprevalence for POWV and EEEV in the counties sampled is generally low. The finding of a low number of infections suggests the risk to the blood supply is minimal in most areas surveyed. However, 2 adjacent counties in Wisconsin had higher POWV seroprevalence than the others, suggesting a potential risk for blood donor infection, although the estimates were imprecise because of small numbers of blood donor samples available from those locations.

Limited data are published on human seroprevalence for POWV and EEEV. For POWV, in a household survey conducted after a 2019 cluster of POWV disease cases in a focal area of New Jersey, estimated neutralizing antibody seroprevalence was 1.1% (95% CI 0%–2.3%) and IgM seroprevalence was 0.31% (95% CI 0.04%–1.0%) (*11*). Those estimates fell within the range of our county estimates for the 2 East Coast states in this study, Connecticut and Massachusetts, despite differences in methodologies and locations sampled. However, we did find higher POWV seroprevalence estimates in some Wisconsin counties. Geographic variation in occurrence and seroprevalence has been well documented for vectorborne diseases and is likely dependent on several factors, such as vector density, infection prevalence in vectors and animal reservoirs, climate effects on ecology, and human behavior affecting a person’s risk for infection (*24*). Further study is warranted to obtain a more precise estimate of POWV seroprevalence in these counties in Wisconsin, the state of residence for the blood donor implicated in the only reported transfusion-transmitted POWV infection (*5*), to determine whether risk for infection might be heightened in that region.

The imprecision in the POWV estimates, particularly in the Midwestern states, precluded us from calculating the proportion of infections that resulted in neuroinvasive disease. Vahey et al. (*11*) reported that 23% (95% CI 7%–100%) of POWV infections result in neuroinvasive disease. That estimate is higher than those for WNV, in which neuroinvasive disease develops in <1% of infected persons (*25*–*27*).

Although we sampled from EEEV-endemic areas affected by the 2019 multistate EEEV disease outbreak (*10*), we found only 1 positive blood donor, for a county seroprevalence estimate of 1.6%. This finding was slightly lower but within the range of the only known published estimate of 2.3% (range by township 0.9%–6.2%) from a 1959 EEEV outbreak in New Jersey, despite differences in serologic methods used (*12*). The finding of just 1 blood donor with antibodies to EEEV after the large outbreak in the season before the samples were collected suggests that human infections are uncommon and the risk to the blood supply is limited.

Our estimate of the percentage of EEEV-infected persons who develop neuroinvasive disease was lower than that of the 1959 New Jersey study, which estimated 4.4% and ranged from 2% in younger adults to 13% in young children (*12*,*28*). Differences in the setting and methodologies of the studies make comparisons of estimates challenging; however, the upper limit of our 95% CI (1.1%) supports the approximation that <5% of EEEV infections result in neuroinvasive disease, although the risk varies by age group (*12*,*28*–*30*). Additional seroprevalence studies conducted after an outbreak could be done to calculate more precise estimates.

The first limitation of our study is that use of a convenience sample of blood donations collected by a single collection agency resulted in small sample sizes in some areas, limiting the precision of the estimates and our ability to assess all areas endemic for these viruses. The lack of blood donor samples from endemic areas such as New Jersey or New York precluded estimates and comparisons in those areas. Also, sample size calculations were based on the only known seroprevalence estimates from focal New Jersey outbreaks (*11*,*12*), which might have led to undersampling in some areas.

The blood donor population is not likely to be representative of the general population in all respects. In addition, the blood donor samples used for this study were collected during the early part of the COVID pandemic, which might have affected the characteristics of the donor population; donors in March 2020–February 2022 were more likely to be repeat donors who were older, white, and women than were donors in the previous 2 years (*31*). However, those demographic factors are not known to be associated with risk for arboviral disease. To address that limitation, we used similar weighting methods to other published studies to generate population estimates from blood donor seroprevalence (*19*,*25*,*32*). Our estimates are similar to those of previous household seroprevalence surveys for the target viruses (*11*,*12*), suggesting our results are plausible.

By testing for antibodies, we cannot directly determine the chance of viremia being present in a blood donor for these pathogens. However, the overall low occurrence of antibodies, including IgM, suggests that having a viremic blood donor would be even less common; for other arboviruses, IgM can persist for months to years after infection, and viremia is present for up to 2 weeks (*21*,*33*,*34*). In addition, studies of WNV-infected blood donors suggest that viremic donors with IgM are less likely to be infectious than those without IgM (*21*,*35*). Consistent with that hypothesis, in the only report of POWV transmission through blood transfusion, the implicated donation was RNA positive and IgM negative (*5*). The duration of IgM persistence in POWV and EEEV is unknown. Although nucleic acid testing could have identified potentially infectious donors, given the low seroprevalence of infection, the likelihood of detecting RNA in an asymptomatic person would have been so low as to require a much larger sample size. Finally, we could have underestimated the seroprevalence of POWV, given that >50% of the flavivirus-positive specimens could not be differentiated between POWV and WNV.

In conclusion, in POWV- and EEEV-endemic areas of the United States sampled during 2019–2020, seroprevalence estimates for POWV and EEEV infection were generally low, suggesting a low risk for transmission by blood transfusion or organ transplantation. Further studies in the Wisconsin counties with higher seroprevalence estimates using high-throughput molecular assays and larger sample sizes could lead to improved understanding of risk. Potential blood donors could lower their risk for tick and mosquito bites by taking such steps as wearing long sleeves and pants, using Environmental Protection Agency–registered insect repellent, and treating clothing and gear with permethrin. Of note, POWV can be transmitted within 15 minutes of tick attachment, so preventing ticks from attaching and removing them before attachment is key (*36*). CDC will continue to work with partners to monitor infectious threats to blood transfusions and organ transplantation and identify prevention and control interventions to reduce the risk among transfusion and transplant recipients.
